# Identification of Risk Factors for Intravenous Infiltration among Hospitalized Children: A Retrospective Study

**DOI:** 10.1371/journal.pone.0158045

**Published:** 2016-06-28

**Authors:** Soon Mi Park, Ihn Sook Jeong, Seong Sook Jun

**Affiliations:** 1 Department of Nursing, Pusan National University Yangsan Hospital, Yangsan, South Korea; 2 College of Nursing, Pusan National University, Yangsan, South Korea; Nottingham University, UNITED KINGDOM

## Abstract

This retrospective study was aimed to identify risk factors of intravenous (IV) infiltration for hospitalized children. The participants were 1,174 children admitted to a general hospital, who received peripheral intravenous injection therapy at least once, and had complete records. Data were analyzed with frequency and percentage or mean and standard deviation were calculated, and odds ratio (OR) from univariate and multiple logistic regressions. The number and % of infiltrations were 92 and 7.8%, respectively. IV infiltration risk factors were lower limb (OR = 1.72), phenytoin (OR = 11.03), 10% dextrose (OR = 6.55), steroids (OR = 6.21), vancomycin (OR = 4.10), high-concentration electrolytes (OR = 3.49), and ampicillin/sulbactam combination (OR = 3.37). Nurses working at children’s hospitals should consider the risk of IV infiltration for children receiving IV infusion therapy and make a preventive effort to identify IV infiltration in high-risk children at an early stage.

## Introduction

Peripheral intravenous (IV) catheter insertion is a common and universal medical practice to provide therapeutic IV medication [1,2]. IV infiltration and extravasation (ie., infusion leaking out of the blood vessel), are frequently observed in the clinical setting as complications related to intravenous injection [3–8]. IV infiltration can lead to problems like discomfort, the need for reinsertion of the intravenous catheters [7,9], or compartment syndrome [10], which can increase not only the period of hospitalization and medical expenses for treatment [11,12] but also permanent damage in children [13]. Considering these negative consequences of IV infiltration, it is important to prevent these outcomes. IV infiltration related factors must, therefore, be identified to determine high-risk groups and come up with appropriate management strategies.

Previous studies on the risk factors for IV infiltration simply described characteristics of children in which IV infiltration occurred, and they were based on small sized on samples, and they were uncontrolled trials or case reports [14]; therefore, they failed to evaluate risk factors based on the comparison of characteristics between the IV infiltration and non-infiltration groups [3,15–17]. In addition, these studies only took into account drug-related factors when exploring the causes of IV infiltration [3,15–17] rather than comprehensively considering physiological or device-related factors.

Therefore, the purpose of this study was to identify the physiological, device, and drug-related factors reported as to whether they have a relationship with IV infiltration occurrence using a large number of samples including children with and without IV infiltration.

## Materials and Methods

### Study Design

This investigation was a retrospective cohort study of children who did or did not experience IV infiltration after receiving an intravenous infusion during hospitalization at a children's hospital.

### Study Subjects

The inclusion criteria were children hospitalized in three wards of a general hospital in Y city, South Korea, who received peripheral intravenous injection therapy at least once, and had complete records. Among a potential sample of 1758 children from August 1 to October 30, 2011, 1174 (66.8%) subjects met the inclusion criteria.

### Study Instrument

One author developed a data collection tool which was named as 'Record on peripheral intravenous injection'. The tool consisted of four parts based on the existing literatures [5,14,18–22]: physiological, device, and drug-related factors along with the occurrence of IV infiltration. Physiological factors included gender, age, height, weight, and medical department of the subjects. Device-related factors included retention time of the intravenous injection, catheter size, and insertion site. Drug-related factors included the type of infusion and injection administered. Retention time for the intravenous injection was calculated by subtracting the starting time from the removal time. Gauge of the intravenous injection needle inserted was recorded as the catheter size, and the insertion site was noted as being in the upper or lower limb. The administered infusions consisted of 5% dextrose, 10% dextrose, 1:4 dextrose solution (SD), saline solution, total parenteral nutrition, amino acids, and lipids. All drugs administered via intravenous injection from the insertion date until the removal date were recorded, and included antibiotics, antiviral agents, 15% mannitol, blood vessel modulating agents, high-concentration electrolytes, anticancer drugs, anticonvulsant reagents, and antitussive compounds. This tool was measured for content validity by one professor of nursing and three head nurses who had worked for over 10 years in children's hospitals, and was shown to have an over 85% item content validity index, and 90% scale content validity index by universal agreement method.

The stage of IV infiltration was scored on a scale of 0 to 4 by using the criteria of Flemmer and Chan [23], where 0 indicated ‘no IV infiltration’ while 1 to 4 indicated the extent of effusion of fluid that had occurred.’ Because it was not clear from their criteria what defined the size of swelling as slight, moderate, and severe, we defined the size of swelling as slight when it was less than 1 inch, moderate when it was 1 to 6 inches, and severe when it was greater than 6 inches according to the Infusion Nurses Society [4] criteria.

### Study Procedure

This study was conducted after receiving approval from The Pusan National University Yangsan Hospital Institutional Review Board (IRB no. 05-2013-029). The obtaining informed consent was waived because of a retrospective study without collecting personal identifiers. To identify the risk factors, one author collected data using study instrument. If a subject received peripheral intravenous injection therapy two or more times during the hospitalization period, data from the first round of therapy were collected. Device-related factors and drug-related factors were investigated from the start of intravenous catheter insertion to occurrence of IV infiltration for subjects with IV infiltration and to catheter removal for subjects without IV infiltration.

### Data Analysis

The collected data were analyzed using SPSS/WIN software (ver. 18.0). A two-tailed test was performed with a significance level (α) of 0.05. A univariate logistic regression analysis was performed to examine the physiological, device, and drug-related factors associated with IV infiltration. Prior to analysis, explanatory variables were generated or newly categorized. For instance, age was classified according to the pediatric classification system [24] as under 1 year of age (infancy), 1–5 years of age (early childhood), 6–10 years of age (school age), and 11–18 years of age (adolescence). Subjects 2 years of age or younger were classified as underweight when the body weight percentile according to gender and age was 5 percentile or lower and overweight when the percentile was 95 percentile or higher. Subjects over 2 years old were classified as underweight when the body mass index percentile according to gender and age was 5 percentile or lower and overweight when the percentile was 95 percentile or higher [24].

For the medical department category, cranial nerve, allergy, respiratory system, digestive system, kidney / internal secretion, heart, and hematologic tumors were grouped into the pediatric medicine department, and general surgery or otorhinolaryngology grouped into the surgery department.

A general drug classification system was followed to evaluate drug factors. Saline solution, 5% dextrose, and 1:4 SD were classified as isotonic infusions while 10% dextrose, total parenteral nutrition, amino acids, and lipids were classified as hypertonic infusions. Among the injections administered, vesicant [25], nafcilin, and ampicillin / sulbactam combinations regarded as moderate IV infiltration risk drugs [26] were classified as penicillin. Cefotaxime that has a risk of wide IV infiltration into blood vessel tissues and tissue damage [21] was separately classified. Dopamine and dobutamine were classified as vasopressors. Calcium gluconate, sodium bicarbonate, potassium chloride, and magnesium sulfate were classified as high-concentration electrolytes.

Finally, a stepwise multiple logistic regression analysis was done to identify the risk factors of IV infiltration. Explanatory variables were significant factors by the univariate analysis.

## Results

### Characteristics of the Subjects

The majority (61%) of the study subjects were males with a mean age of 6.3 years and 66.6% of them had a normal body weight. Less than half (40%) of the subjects received treatment for allergies and respiratory system problems (Table 1).

**Table 1:** Physiologic Factors Related to Intravenous Infiltration.

Factors	N(%)	Occurrence of infiltration n (%)	Odds ratio (OR)	95%CI	*p*
Gender					
Male	711(61)	53(7.5)	1.00		
Female	463(39)	39(8.4)	1.14	0.74–1.76	0.5
Age (years)					
<1	83 (7)	7(8.4)	1.07	0.43–2.64	0.8
1~5.9	589(51)	45(7.6)	0.96	0.55–1.68	0.8
6~10.9	260(22)	21(8.1)	1.02	0.53–1.94	0.9
≥11	239(20)	19(7.9)	1.00		
Nutritional status					
Underweight	300(26)	32(10.7)	1.61	1.01–2.54	0.043
Normal	780(66)	54(6.9)	1.00		
Obese	92(8)	6(6.5)	0.94	0.39–2.25	0.9
Medical department					
Pediatric medicine	892(76)	84(9.4)	1.00		
Surgical	282(24)	8(2.8)	0.28	0.13–0.59	0.001

Mean retention time of the intravenous injection was 59.96 hours. The size of 94% of the catheters used was 24-gauge. The insertion site was in the upper limb for 85% of the subjects (Table 2).

**Table 2:** Device-related Factors Related to Intravenous Infiltration.

Factors	N(%)	Occurrence of infiltration n (%)	Odds ratio (OR)	95%CI	*p*
Retention time (hours)					
≤24.0	203(17)	13(6.4)	1.00		
24.1–48.0	319(27)	28(8.8)	1.41	0.71–2.78	0.3
48.1–72.0	378(32)	26(6.9)	1.08	0.54–2.15	0.8
72.1–96.0	230(20)	18(7.8)	1.24	0.59–2.60	0.6
96.1–120	44 (3)	4(11.1)	1.83	0.56–5.96	0.3
≥120.1	10 (1)	3(37.5)	8.77	1.88–40.81	0.006
Catheter size(gauge)					
≤22	70 (6)	1(1.4)	0.16	0.02–1.18	0.072
24	1104(94)	91(8.2)	1.00		
Insertion site					
Upper limb	992(85)	65(6.6)	1.00		
Lower limb	181(15)	27(14.9)	2.50	1.55–4.04	<0.001

The largest portion of subjects (64%) received an infusion of 5% dextrose while 27% of the subjects were treated with cefotaxime (Table 3).

**Table 3:** Drug-related Factors Related to Intravenous Infiltration.

Factors	N(%)	Occurrence of infiltration n(%)	Odds ratio (OR)	95%CI	*p*
Fluid administered					
10% Dextrose	33(3)	7(21.2)	3.35	1.41–7.93	0.004**
5% Dextrose	754(64)	67(8.9)	1.54	0.96–2.48	0.073
Lipid	9(1)	2(22.2)	3.41	0.70–16.67	0.1
TPN	45(4)	6(13.3)	1.87	0.77–4.53	0.2
Normal saline	30(3)	4(13.3)	1.85	0.63–5.41	0.2
1:4 SD (0.225% NS)	210(18)	16(7.6)	0.96	0.55–1.69	0.8
Amino acid	8(1)	0(0.0)	0.00	0.000-	0.9
Drug administered					
Phenytoin	10(1)	5(50.0)	12.38	3.52–43.59	<0.001***
Ampicillin/sulbactam combinations	103(9)	21(20.4)	3.61	2.11–6.17	<0.001***
Cefotaxime	317(27)	38(12.0)	2.03	1.31–3.14	0.002**
High-concentration electrolytes	18(2)	5(27.8)	4.73	1.65–13.56	0.004**
Vancomycin	15(1)	4(26.7)	4.43	1.38–14.19	0.012*
Steroids	30(3)	6(20.0)	3.08	1.22–7.73	0.017*
Aminoglycoside	20(2)	4(20.0)	3.03	0.99–9.25	0.052
Hemocoagulase	65(6)	1(1.5)	0.18	0.02–1.28	0.085
Immunoglobulin	8(1)	2(25.0)	3.99	0.79–20.03	0.093
Acylclovir	2(0.2)	1(50.0)	11.88	0.74–191.49	0.1
Nafcillin	5(0.4)	1(20.0)	2.96	0.33–26.77	0.3
15% mannitol	58(5)	7(12.1)	1.67	0.73–3.78	0.3
Non-steroid anti-inflammatory drugs	63(5)	3(4.8)	0.57	0.18–1.87	0.3
Ambroxol	583(50)	44(7.5)	0.92	0.60–1.41	0.7
Vasopressors	10(1)	1(10.0)	1.31	0.16–10.46	0.8

SD = dextrose solution

* p<0.05,

** p<0.01,

*** p<0.001

### Risk factors for IV infiltration

According to the univariate analysis, physiological factors related to the occurrence of IV infiltration were being underweight (Table 1). Patients admitted to the surgical ward were less likely to have infiltration. Significant device-related factors were retention time and insertion site (Table 2). Significant drug-related factors were 10% dextrose, use of medicines such as phenytoin, high-concentration electrolytes, vancomycin, ampicillin/sulbactam combinations, steroids, and cefotaxime (Table 3).

Multivariate analysis confirmed the findings on univariate analysis with the exception of being underweight, retention time, and use of cefotaxime. Results of the analysis identified insertion site, medical department admission, phenytoin, 10% dextrose, steroids, vancomycin, high-concentration electrolytes, and ampicillin/ sulbactam combinations as significant risk factors (Table 4).

**Table 4:** Intravenous Infiltration Risk Factors by Multiple Logistic Regression.

Factors	B	SE	OR	95%CI	*p*
Phenytoin	2.400	0.678	11.03	2.92–41.68	<0.001
10% Dextrose	1.879	0.522	6.55	2.36–18.20	<0.001
Steroids	1.826	0.568	6.21	2.04–18.91	0.001
Vancomycin	1.410	0.638	4.10	1.17–14.30	0.027
High-concentration electrolytes	1.250	0.578	3.49	1.13–10.82	0.031
Ampicillin/sulbactam	1.215	0.289	3.37	1.91–5.94	<0.001
Insertion site(lower limb)	0.544	0.270	1.72	1.02–2.92	0.044

SE: standard error, OR: odds ratio, CI: confidence interval

The ORs for use of phenytoin and 10% dextrose were 11.03 (95% CI = 2.92–41.68, *p <*0.001) and 6.55 (95% CI = 2.36–18.2, *p <* 0.001), respectively. The OR for pediatric medicine admission was 3.97 (95% CI = 1.68–9.36, *p* = 0.002), and the OR for the lower limb as insertion site was 1.72 (95% CI = 1.02–2.92, *p* = 0.044).

## Discussion

Risk factors of IV infiltration were lower limb insertion site, pediatric medicine admission, and administration of phenytoin, 10% dextrose, steroids, vancomycin, high concentration electrolyte, ampicillin/sulbactam combinations in this study.

Certain fluids and drugs can easily cause venous rupture when the venous endothelium and blood vessel walls are irritated, leading to IV infiltration or extravasation. Since strong acids or strong alkalinities can damage cellular proteins, reduce durability of the venous endothelium, and inflict venous rupture, pH ranging from 5 to 9 is recommended for intravenous injection [14]. Phenytoin is as an anticonvulsant that is also a strong alkaline [27] with a pH of 12, and has been found to reduce vein durability by damaging cell proteins [21,26]. Vancomycin is a strong acid with a pH of 2.8–4.5 [27], and a study has shown several cases who developed infiltrations after the administration of vancomycin [28]. Ampicillin / sulbactam combinations have strong alkalinity with a pH of 8–10, and cause irritation to the vessel walls and facilitate the occurrence of infiltration [26]. Previous studies have reported total skin loss after administration of penicillin antibiotics [15].

Likewise, osmotic pressure within the range of 250–350 mOsm/L is permitted to be given peripherally [14,27]. High osmotic pressure exceeding 350 mOsm/L can disrupt cell functions and rupture cells according to movement of moisture from cell to interstitial space [14]. Osmotic pressure above 600 mOsm/L causes peripheral phlebitis within 24 hours in a study [4]. On the other hand, low osmotic pressure below 250 mOsm/L results in rupture of cells due to edema [14]. High-concentration electrolytes have high osmotic pressure and have strong alkalinity that can damage the vein [14,21,26], resulting in an increased risk of IV infiltration. Previous studies have reported significant local tissue necrotic cases caused by peripheral extravasation of intravenous solutions containing high-concentration electrolytes like calcium salts [16,29]. A 10% dextrose solution identified as a risk factor of IV infiltration in this study is associated with high osmotic pressure of 505 mOsm/L [18,26]. Hyperosmolar agents such as 10% dextrose or total parenteral nutrition solutions can cause skin necrosis and serious issue damage [14,18] and facilitate the occurrence of infiltration. Yosowitz et al [29] have reported 8 patients, including 6 infants who had significant local tissue necrosis by peripheral extravasation of intravenous solutions containing calcium salts and/or 10% dextrose.

Steroids are known to induce the formation of emboli when used over a long period of time [30]. Emboli frequently arise due to repeated damage of blood vessels and coagulation activated by platelets and fibrin. When embolus formation is continued, blood flow is affected and blood vessel walls are weakened becoming vulnerable to IV infiltration [31].

According to the results, nurses in clinical settings are recommended to select an IV site in upper arm rather than in lower limb, and need to make repeated observations to identify IV infiltration occurrence when they care for children in the pediatric medicine department, and administer high risk drug/fluid such as phenytoin, 10% dextrose, steroids, vancomycin, high-concentration electrolytes, and ampicillin/sulbactam.

To our knowledge, this study is one of the few studies to identify the risk factors of IV infiltration considering different factors in a large group of subjects, divided into children with and without IV infiltration. However, careful interpretation of the results from the present investigation is required as this study has the following two limitations. First, there is limitation in generalizability because the study only included children hospitalized at a single children's hospital. Second, this study was a retrospective survey of medical records and thus failed to reflect characteristics used by the nurses to determine IV infiltration risk factors. This includes factors such as catheter insertion skill and appropriateness of catheter fixation after insertion.

## Conclusions

Risk factors related to the occurrence of IV infiltration in children were assessed in the present study. Based on the findings, nurses working at the children’ hospitals should consider the risk of IV infiltration for children receiving intravenous infusion therapy and make efforts to identify IV infiltration in high-risk children at an early stage to prevent damage. Furthermore, a replication study on children with different characteristics should be conducted to increase the possibility for generalization of the risk factors. A prospective study should also be performed to investigate the nurse-associated factors such as catheter insertion skill.
